# Friction Stir Processing of Particle Reinforced Composite Materials

**DOI:** 10.3390/ma3010329

**Published:** 2010-01-11

**Authors:** Yong X. Gan, Daniel Solomon, Michael Reinbolt

**Affiliations:** 1 Department of Mechanical, Industrial and Manufacturing Engineering, College of Engineering, University of Toledo, 2801 W Bancroft Street, Toledo, OH 43606, USA; 2 Department of Mechanical Engineering, Albert Nerken School of Engineering, The Cooper Union for the Advancement of Science and Art, 41 Cooper Square, New York, NY 10003, USA

**Keywords:** friction stir processing, particle reinforcements, composite materials

## Abstract

The objective of this article is to provide a review of friction stir processing (FSP) technology and its application for microstructure modification of particle reinforced composite materials. The main focus of FSP was on aluminum based alloys and composites. Recently, many researchers have investigated this technology for treating other alloys and materials including stainless steels, magnesium, titanium, and copper. It is shown that FSP technology is very effective in microstructure modification of reinforced metal matrix composite materials. FSP has also been used in the processing and structure modification of polymeric composite materials. Compared with other manufacturing processes, friction stir processing has the advantage of reducing distortion and defects in materials. The layout of this paper is as follows. The friction stir processing technology will be presented first. Then, the application of this technology in manufacturing and structure modification of particle reinforced composite materials will be introduced. Future application of friction stir processing in energy field, for example, for vanadium alloy and composites will be discussed. Finally, the challenges for improving friction stir processing technology will be mentioned.

## 1. Introduction

**Figure 1:**
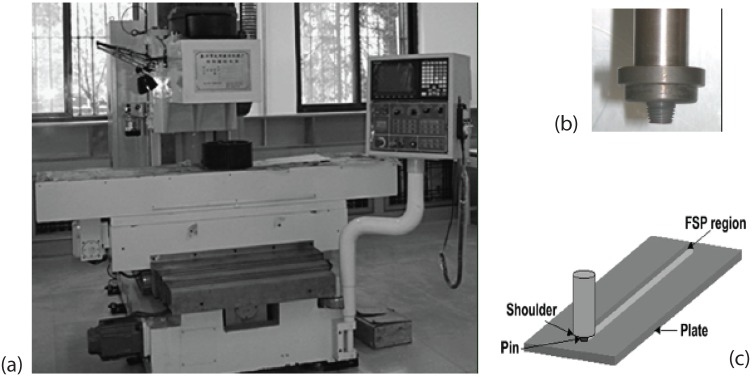
Equipment for friction stir processing: (a) picture of friction stir processing machine (after Wang *et al.* [3]); (b) tool pin (after Ceschini *et al.* [11]); (c) schematic showing the principle of friction stir processing (after Mishra *et al.* [12]).

Friction stir processing (FSP) is a solid state process known for its ability to modify microstructures and provide improved properties over conventional processing technologies [1,2,3,4,5,6,7,8,9,10,11,12]. The development of friction stir processing (FSP) is based on the friction stir welding (FSW) technology. Both FSW and FSP have the same process principle and share the same facilities as shown in Figure 1(a). FSW works by plunging a spinning tool into the joint of two materials and then traversing the rotating tool along the interface. The friction caused by the tool heats up the materials around the pin to a temperature below the melting point. The rotation of the tool “stirs” the material together and results in a mixture of the two materials. In FSP, a specially designed rotating pin, as shown in Figure 1(b), is first inserted into the material to be processed with a proper tool tilt angle and then move along the designed paths. The pin produces frictional and plastic deformation heating within the processing zone. As the tool pin moves, materials are forced to flow around the pin. Material flows to the back of the pin, where it is extruded and forged behind the tool, consolidated and cooled under hydrostatic pressure conditions. It is evident that FSW and FSP share the same mechanism as schematically illustrated in Figure 1(c). That is why in some literatures, the uses of the two terms are interchangeable. However, they do have different purposes in practical applications. The goal of FSW is to join two plates together, whereas FSP aims at modifying the microstructure of a single workpiece or multiple workpieces.

Friction stir processing is most commonly used with aluminum [13,14,15,16,17,18,19,20,21,22], but it is also used for processing other alloys, for example, Ni-based intermetallic compound modified bronzes [23], Zr alloys [24], tool steels [25,26] and Mg alloys [27,28,29,30,31,32,33]. The advantages offered by friction stir processing method make it suitable for many applications. Recently, friction stir processing technology has been used in aerospace, automotive, marine, and railroad industries along with various other applications. Experimental [34,35,36,37,38,39,40,41,42,43], analytical [44,45] and computational studies of friction stir processing nanocomposites [46,47] have been performed. Specifically, the effects of processing parameters on microstructure evolution, deformation behaviors and mechanical properties are investigated [48,49,50,51,52].

## 2. Friction Stir Processing of Aluminum Matrix Composites

Friction stir processing has been used in various type of particle reinforced aluminum composite materials. SiC particle reinforced aluminum alloys have been studied the most [25,43,53,54,55,56]. For example, the feasibility of making bulk ${\mathrm{SiC}}_{p}$ reinforced aluminum metal matrix composites (MMCs) with the dimension of 150 mm in length, 60 mm in width and 6 mm in depth via friction stir processing (FSP) was demonstrated by Wang *et al.* [3]. Good interface bonding between particles and the base metal can be obtained. The volume percentage of ${\mathrm{SiC}}_{p}$ is about 1.5% in the reinforced region. The microhardness of the reinforced MMCs is 10% higher than that of the base metal, Al-6Mg-Mn.

**Figure 2:**
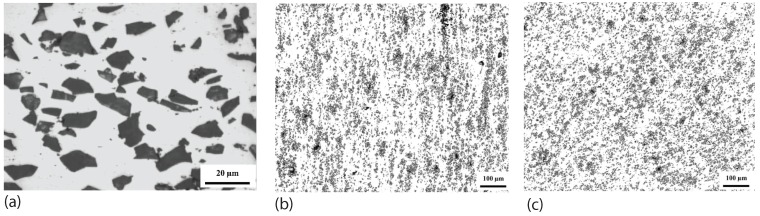
Microstructure change in SiC particle reinforced A6061 due to friction stir processing (after Tewari *et al.* [53]): (a) scanning electron microscopic image of the composite showing the anisotropic shape of SiC particles; (b) as-extruded SiC/Al; (c) after FSP.

Tewari *et al.* [53] studied the effect of SiC particle orientation change due to friction stir processing. High-resolution, large-area images were obtained to show the detailed microstructural data for SiC/A6061 composite materials, both before and after single friction stir processing step. The SiC reinforcement particles were found to have anisotropic shape with an average aspect ratio around 1.6 to 1.8. The scanning electron microscopic image of the composite in Figure 2(a) shows such a morphological feature. From particle orientation statistics, it was found that there are preferred orientations for the nonequiaxed SiC particles after extrusion processing. As shown in Figure 2(b), the alignment of the SiC particles is parallel to the extrusion direction. The extrusion axis is vertical. The preferred orientation can be modified during passage of the friction stir tool. In Figure 2(c), the redistributed particles at $45^{\circ }$ to the extrusion and transverse directions can be seen. The FSP tool motion is horizontal, left to right. The microstructural data consistently indicate that significant microstructural modifications occur during FSP, including re-orientation of the reinforcement particles, and a significant reduction in the levels of microstructural heterogeneity and microstructural anisotropy.

**Figure 3:**
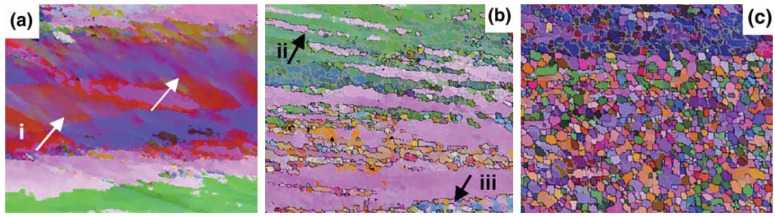
Electron backscatter diffraction (EBSD) maps showing the development of grain structures in different zones associated with the strain and temperature change in friction stir processing (after Prangnell and Heason [57]): (a) materials far away from the tool pin showing split grains into deformation bands; (b) materials close to the tool pin showing parallel bands separated by fine grains; (c) very fine nugget grains in the material immediately next to the tool pin surface.

The grain structure change in friction stir processed materials has caught considerable attention. Prangnell and Heason [57] investigated the microstructure development in selected zones. What they found is that the deformation field surrounding the rotating pin caused the formation of the ultrafine grained nugget material. In the friction stir processing zone far away from the tool pin, the grains can be seen splitting into deformation bands due to the flow of the Al4.8Cu0.9Li alloy as marked by **i** in Figure 3(a). In the zone closer to the tool pin, increase in misorientation and reduce in spacing of the grains were found as marked by **ii** at the upper left corner in Figure 3(b). Finely spaced, parallel bands were also found and they broke up along their length by thermally and mechanically assisted boundary migration to form fine nugget-scale grains marked by **iii** at the lower right corner in Figure 3(b). In Figure 3(c), the enlarged fine nugget-scale grains are shown. The material is immediately next to the pin surface.

**Figure 4:**
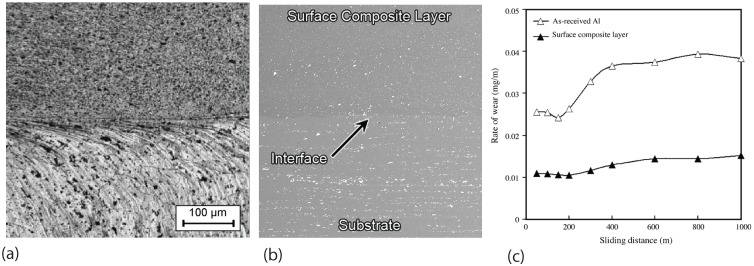
Microstructure and wear property of friction stir processing alumina particle reinforced A6082 aluminum alloy (after Shafiei-Zarghani *et al.* [9]): (a) optical micrograph showing the composite top layer and the bottom layer of matrix alloy; (b) SEM image showing the well bonded interface between the two layers; (c) comparison of wear rate of the as-received alloy and the material with a surface nano-composite layer produced by four FSP passes.

Friction stir processing alumina particle reinforced aluminum alloys has also been studied [9,58,59,60,61]. Shafiei-Zarghani *et al.* [9] used friction stir processing (FSP) to incorporate nano-sized Al_2_O_3_ into AA6082 aluminum alloy to form particulate composite surface layer as shown in Figure 4(a). The Al_2_O_3_ particles have an average size of about 50 nm. Perfect bonding between the surface composite and the aluminum alloy substrate was achieved as shown by the defect-free interface in Figure 4(b). Mechanical properties include microhardness and wear resistance were tested. The results show that the increasing in number of FSP passes causes more uniform distribution of nano-sized alumina particles. The microhardness of the surface improves by three times as compared to that of the as-received Al alloy. A significant improvement in wear resistance in the nano-composite surfaced Al was observed as compared to the as-received Al alloy. The wear rate is reduced to one third of that of the as-received Al alloy when friction stir processed composite layer was incorporated as shown by the curves in Figure 4(c).

**Figure 5:**
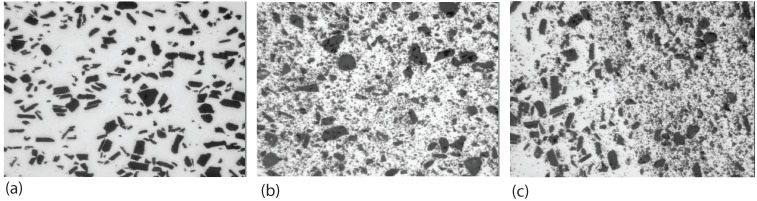
Effect of friction stir processing on the distribution of alumina particles in 6061 aluminum alloy (after Marzoli *et al.* [60]): (a) micrograph showing the composite before FSP; (b) micrograph showing the composite after FSP; (c) microstructure transition near the nugget zone.

Similar to SiC reinforced aluminum composites, the stirring of the FSP tool has a substantial influence on the distribution of alumina particles in Al_2_O_3_/Al composites [60]. The stirring of the tool can also change the shape of the reinforcement particles. It breaks off the sharp edges of the bigger particles, rounding them up at the same time [61]. This action results in smaller, round particles in the nugget, which can be seen from the micrographs in Figure 5. Figure 5(a) represents the morphology of the Al_2_O_3_ particle reinforced AA6061 before FSP. Sharp corners of the particles are very clearly shown. After FSP, scattering in the size distribution of the Al_2_O_3_ particles within the composite material were more pronounced, which can be easily found from the morphological features shown in Figure 5(b). Many small particles are around those big-sized ones. This is due to the fracture of the original Al_2_O_3_ particles. In addition, some sharp Al_2_O_3_ particles became rounded. Structure transition was also observed in the area close to the nugget as shown in Figure 5(c).

**Figure 6:**
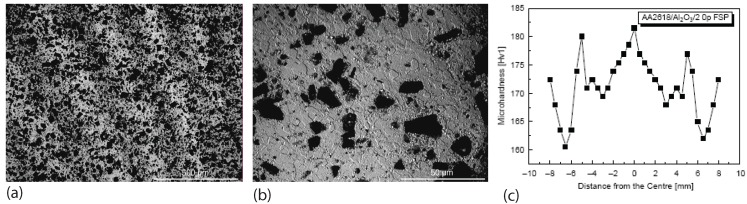
Different zones generated by friction stir processing as revealed by morphology and microhardness profile (after Cavaliere [61]): (a) micrograph showing the deformation bands in TMAZ; (b) recrystallization in nugget zone; (c) microhardness profile in the FSP Al_2_O_3_/AA2618 composite.

Macrostructure observations on friction stir processed Al_2_O_3_ particle reinforced aluminum composites revealed three distinct zones, i.e. the heat affected zone (HAZ), thermomechanically affected zone (TMAZ) and dynamically recrystallized nugget zone. The microstructure change in HAZ is less obvious. However, particle fracture was found in this zone as indicated by Cavaliere [61]. In the thermomechanically affected zone (TMAZ), deformation bands was observed as shown in Figure 6(a). In the nugget zone, recrystallized microstructure and fractured particles are the major morphological features as illustrated by Figure 6(b). There exists difference in microhardness of these zones. As shown in Figure 6(c), the microhardness for the material in the nugget zone is the highest because the grain size is the smallest in this zone.

**Figure 7:**
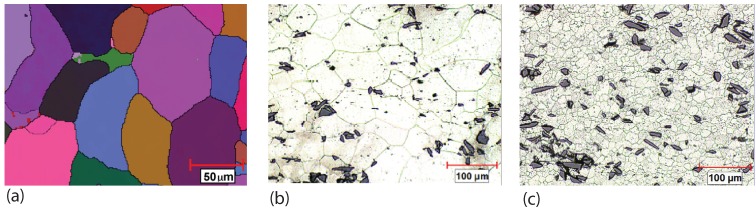
AA6063 and B_4_C particle reinforced composite (after Chen *et al.* [62]): (a) EBSD orientation map showing the grains of AA6063; (b) B_4_C particle reinforced composite before FSP; (c) B_4_C particle reinforced composite after FSP.

In addition to SiC and Al_2_O_3_ particles, B_4_C particles were used to make discontinuously reinforced aluminum composite. The structures of the composite with AA6063 matrix reinforced by 6 and 10.5 vol % B_4_C particles were modified by friction stir processing [62]. The EBSD orientation map, Figure 7(a), shows the grain structure of the AA6063 aluminium alloy. The presence of B_4_C particles and the volume content did not change the grain structure much. The composite with 6% of B_4_C particles has almost the same equiaxed grain structure as the matrix alloy. In Figure 7(b), the morphology of the composite is shown. After friction stir processing, the refined grains were observed within the composite as revealed by Figure 7(c). This indicates that the grain size of the AA6063 aluminium matrix is reduced considerably in the stirred zone. However, the size and shape of B_4_C particles were not significantly changed by FSP. Rather, the particle distribution in the matrix was more uniform and the number and size of reinforcement particle clusters were reduced in the stirred zone. It was also found that during FSP, dynamic recrystallization happened in the nugget zone. The continuous evolution of aluminium grain structure from coarse grains of the AA6063 matrix alloy via subgrain development occurred within coarse grains to the refined, equiaxed grains in the center of FSP zone.

**Figure 8:**
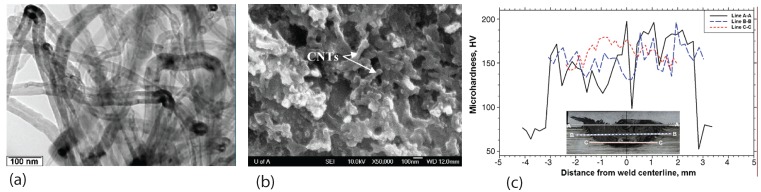
Morphology and hardness profile of friction stir processed carbon nanotube reinforced composite (after Lim *et al.* [14]): (a) TEM image of multi-walled carbon nanotubes; (b) friction stir processed composite; (c) hardness measurement results.

Nanoscale reinforcements such as carbon nanotubes were used to make reinforced composite materials through FSP [14]. The effects of processing parameters on multi-walled carbon nanotube dispersion, and hardness of the composite were investigated. Increasing the tool rotation speed and the tool shoulder penetration depth improved the distribution of nanotubes in the Al-alloy matrices, 6111-T4 and 7075-T6. A completely uniform distribution could not be achieved when regularly tangled nanotubes as shown in Figure 8(a) were used as the starting material. Fracture of carbon nanotubes was found during friction stir processing as revealed by Figure 8(b), the surface morphology of an etched specimen. The etching helps to expose the carbon nanotubes. Evidently, the long and entangled CNTs are not uniformly dispersed throughout the processing zone. Although it is suggested that multiple tool pin passes may be useful to further improve the uniform dispersion of nanotubes in the composites, fracture of carbon nanotubes could be more serious. Nevertheless, carbon nanotube reinforced composites showed the significant strengthening effect, as evidenced by the drastic increase in the hardness in the friction stir processing zone. That the hardness of the processing zone is higher than other regions is shown in Figure 8(c).

**Figure 9:**
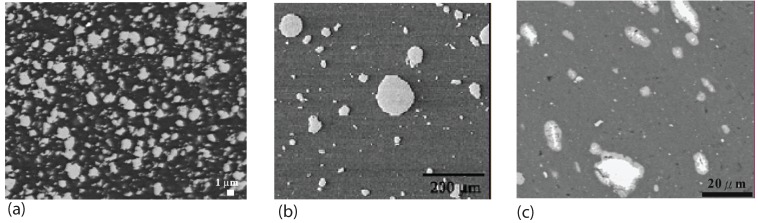
Backscattered electron images showing friction stir processed *in-situ* particle reinforced composite materials: (a) the dispersion of fine Al_2_Cu particles (white contrast) in Al10Cu alloy (after Hsu *et al.* [63]); (b) uniformly distributed NiTi particles in the nugget region of FSP composite after annealing (after Dixit *et al.* [64]); (c) Al_3_Fe particles in Al matrix (after Lee *et al.* [65]).

Other types of micro- or nanoscale phases, such as *in-situ* formed particles including Al_2_Cu, and Al_3_Ti [63], NiTi [64], and AiFe [65] have been taken as the reinforcements for aluminum based composites and FSP has been used to modify the microstructures of these *in-situ* composites. Under high temperature processing conditions such as casting and powder metallurgical formation, considerable interfacial reactions lead to formation of a number of intermetallic phases, such as Al_3_Ti, Al_3_Ni, Ni_3_Ti, Ti_2_Ni, etc. depending on the compositions of the raw materials. The presence of these intermetallic phases, if they are physically or mechanically incompatible with the matrix alloys, they become the key sites for fracture initiation and failure. FSP could help to alleviate this problem. The advantage of *in-situ* composites is that particles are dispersed very uniformly within matrices as shown by the backscattered electron images (BEI) in Figure 9. Figure 9(a) represents the Al_2_Cu particles in Al-10Cu alloy. The spots with white contrast are the Al_2_Cu particles. The distribution of the particles are very uniform. In Figure 9(b), NiTi particles (white contrast) in the nugget zone are shown. Figure 9(c) reveals the Al_3_Fe particles in Al matrix after 2 passes of FSP followed by annealing. The brightest phase is identified as Fe through EDS, the dark gray matrix is Al, and the light gray phase, which surround Fe particle or dispersed in the Al matrix, is AlFe compound.

## 3. Friction Stir Processing of Magnesium Matrix Composites

Magnesium has the density as small as 1.7 g/cm^3^, which is the lowest among all the structural metals or alloys [66]. Such an outstanding property makes it extremely important for manufacturing light weight structures. However, magnesium is very soft. It is necessary to add either alloy elements or reinforcements to make it stronger. Recently, friction stir processing particle reinforced magnesium has been studied [29,30,32,67,68,69,70]. In the work performed by Lee *et al.* [67], Nanoscale SiO_2_ with the dimension of 20 nm was added into an AZ61 magnesium alloy. Friction stir processing (FSP) was applied to incorporate 5-10 vol % nanoparticles into the matrix. The distribution of the SiO_2_ particles was uniform after four FSP passes. The grain size of the FSP composites was effectively refined to 0.8 *μ*m, as compared with the 78 *μ*m in the FSP AZ61 alloy processed under the same FSP condition. There is reaction between SiO_2_ particles and the Mg alloy during FSP. The products from the reaction include Mg_2_Si and MgO. The size of these products is in the range from 5 to 200 nm, which is very fine and good for improving the mechanical properties. The hardness and mechanical strength at room temperature of the AZ61Mg composite with nano-particles were increased, as compared with the AZ61 cast billet. Also found is the high strain rare superplasticity over 400% in the FSP composite.

In the work performed by Morisada *et al.* [68], multi-walled carbon nanotubes (MWCNTs) with the outer diameter of 20 to 50 nm, and length of 250 nm were successfully dispersed into AZ31 magnesium alloy via friction stir processing (FSP). The tool rotating speed was kept at 1500 rpm. Three travel speeds of 100, 50 and 25 mm/min, were used. It was found that the travel speed of 100 mm/min was too fast to produce enough heat flow to produce a suitable viscosity in the AZ31 matrix for the dispersion of the MWCNTs. The sample which was processed at 50 mm/min showed a better dispersion of the MWCNTs. However, there were some regions where aggregation of MWCNTs occurred. A much better dispersion of the MWCNTs was achieved for the sample that was processed at 25 mm/min. The addition of the MWCNTs promoted grain refinement during the FSP. The size of the grains can be controlled as small as 500 nm. The Vickers hardness of the friction stir processed composite material containing MWCNTs is 78 HV. For the sample without MWCNTs treated by the FSP, the measured hardness is 55 HV. The as-received AZ31 sample has the lowest hardness of 41 HV.

**Figure 10:**
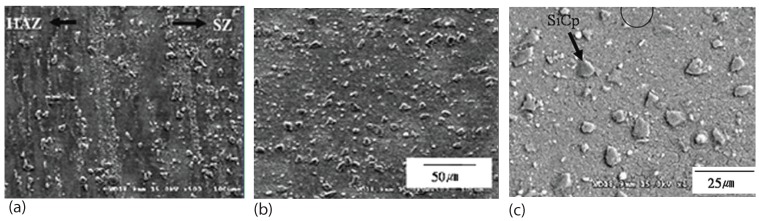
Microstructure of SiC particle reinforced AZ91 magnesium alloy composite material (after Lee *et al.* [69]): (a) the area showing the transition from thermomechanical affected zone (TMAZ) to stir zone (SZ); (b) uniformly distributed SiC particles in the stir zone; (c) higher magnification image of stir zone showing the SiC particles on the etched surface of the composite.

Lee *et al.* [69] studied the microstructure and wear properties of friction stir processed SiC particle reinforced AZ91 magnesium alloy composite material. The composite contains 10 vol % SiC particles. In the thermomechanically affected zone (TMAZ), there are deformation bands and the distribution of SiC particles aligned along the bands as can be seen from Figure 10(a). In the stir zone (SZ), SiC particles are uniformly distributed as shown in Figure 10(b). Concerning the grain structure evolution of magnesium alloy without reinforcement, Suhuddin *et al.* [70] provided detailed results of grain size and orientation to address the twinning, texture formation and recrystallization due to FSP.

At higher magnification as shown in Figure 10(c), it is observed that the sharp corners of SiC particles were rounded up. Before taking SEM images, the surface of the composite was etched for 30 s with a solution containing ethyl alcohol, acetic acid, picric acid and distilled water to reveal the SiC particles. The wear tests for the AZ91, AZ91/SiC/10p and the FSP zone of the AZ91/SiC/10p were performed under wet sliding conditions for 1000 s against a rotating Austenite cast iron disc (hardness 200 HV) at the constant load of 50 N and sliding speed of 1 m/s. The lubricant was distilled water. The specific wear loss, *W*, was used to measure the wear resistance of the materials, and *W* is calculated by the following equation

(1) $$W=\frac{Bb^{3}}{8rPl}$$

where *B* is the thickness of the rotating disk, *r* is the radius of the disk, *b* is the length of the wear trace, *P* is the applied load and *l* is the sliding distance.

**Figure 11:**
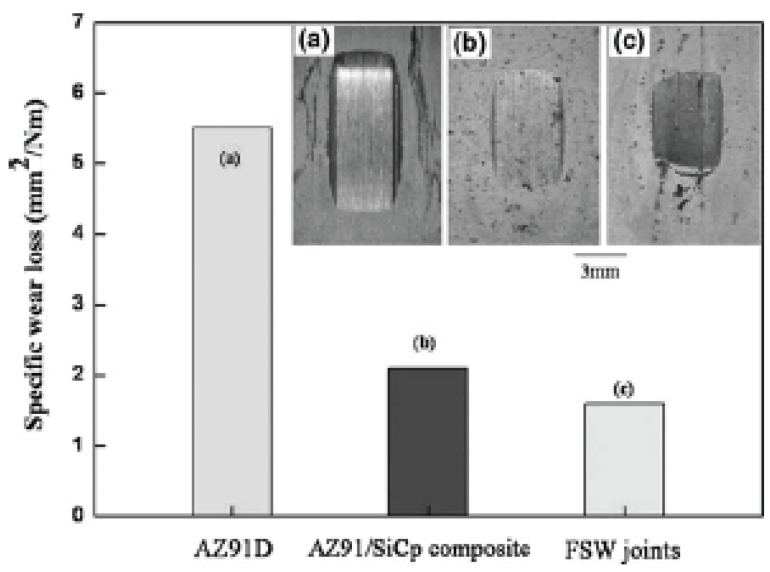
Wear loss of AZ91, SiC/AZ91 composite without and with FSP (after Lee *et al.* [69]).

Based on the wear test data and the calculation using the above Equation (1), the specific wear loss of the base alloy is about three times higher than that of the SiC reinforced composite before FSP. The wear loss of the composite can be further reduced after FSP as shown in Figure 11.

## 4. Perspectives on Friction Stir Processing

### 4.1. Application for Processing New Metallic Alloys/Composites

Friction stir processing is expected to find more applications, for example, in energy field. Perhaps to process or join vanadium alloys and nanoparticle reinforced copper composite materials for fusion reactor applications is such a good example. V-Ti-Cr alloys and nanocomposites made by electrocodeposition of SiC nanoparticles with copper can be joined by optimizing the parameters such as tool rotating speed, nutting angle and feed rate to obtain effective butt joints. Development of fusion reactor technology for power generation is an attractive proposition because of the abundant availability of hydrogen isotope and the fusion process is much safer than the currently used fission technology. The property requirements for fusion structures such as the first wall and divertor are very stringent. The fusion structural materials should have good mechanical properties at high temperatures, good corrosion resistance with the coolant, be able to accommodate high heat loads, and have low levels of induced radioactivity. Vanadium alloys are under consideration for use in fusion energy systems as structural materials because they offer significant advantages over other iron based alloys in several of the areas mentioned above [71,72]. Extensive research in the past two decades has lead to a basic understanding of the physical, mechanical and metallurgical aspects of vanadium alloys including the effects of radiation. Presently, V-Cr-Ti alloys are considered as the promising ones with improved performance [71,72,73,74,75].

The vanadium alloys must be joined to either the vanadium alloys with different configuration or to lower cost dissimilar metallic materials such as copper alloys to construct an effective power generating system. In structural fabrication, such as the first wall and divertor components of a nuclear reactor, kilometers of welds are expected. Harnessing the excellent mechanical properties and environmental safety features offered by vanadium alloys depends upon the successful development of processing technology for vanadium alloys. There exists a technical challenge in conventional welding or brazing vanadium to vanadium or to copper alloys due to their affinity to interstitial elements such as oxygen, hydrogen and nitrogen [76]. At present time, vanadium alloys can be brazed under high vacuum and high temperature conditions. The high vacuum environments can only be created in a laboratory setup, which can be very expensive to maintain. This poses engineering limitations on the size of the structures that can be processed. In addition, the temperature in brazing process is so high that undesired coarse grains formed in both vanadium alloys and the joining counterpart alloys.

Apart from brazing, other joining methods have also been studied to obtain sound vanadium joints. Arc welding such as gas tungsten arc (GTA) welding, laser welding and electron beam welding were tried in fabrication of joints containing vanadium alloys [77,78,79]. However, the rapid heating and cooling created complex non-equilibrium microstructure in the fusion and heat affected zones (HAZ), close to the fusion line. This problem got further worsened by the affinity of vanadium alloys to interstitial elements such as O, H, and N. Safety and reliability of welded structures are major concerns in critical application areas such as nuclear reactors. The consequences of catastrophic fracture in a critical structure are very severe, because it may result in loss of human life, environmental degradation and loss of revenue. Unfortunately, welded structures are more prone to fracture because of the presence of residual stresses, non-equilibrium microstructure, cracks and other metallurgical defects. Vanadium alloy joins produced using gas tungsten arc, laser and electron beam welding have been found to have low fracture toughness due to the embrittlement of heat affected zone [80,81]. It is obvious that current practices used to process vanadium alloy joints are not good enough for engineering applications. Therefore, developing innovative processing technology to achieve high quality vanadium alloy joints for industrial applications is one of the most significant problems to be solved.

Friction stir welding (FSW) [82] and friction stir processing (FSP) have been applied to various materials including plastics [83] stainless steels [84,85], magnesium alloys [86], titanium alloys [87], pure copper [88], copper-zinc alloys [89] and metal matrix composite materials [90]. FSW or FSP has many advantages such as elimination of the defects named crack and porosity often associated with fusion welding processes, reduced distortion. In addition, joint edge preparation is much less strict. It can be carried out in various positions and can join conventionally non-fusion weldable alloys and improve mechanical properties of weldable alloys. FSP has also been used to refine the grains of casting alloys [91], and to homogenize the microstructure of reinforced metal matrix composite materials [92]. FSP has found applications in transportation industries [93,94]. The application of FSP in manufacturing of fusion reactor structures has also been considered [95]. Therefore, the application of FSP/FSW in preparation and structure modification of energy materials is promising.

### 4.2. Application for Processing Polymeric Materials and Composites

**Figure 12:**
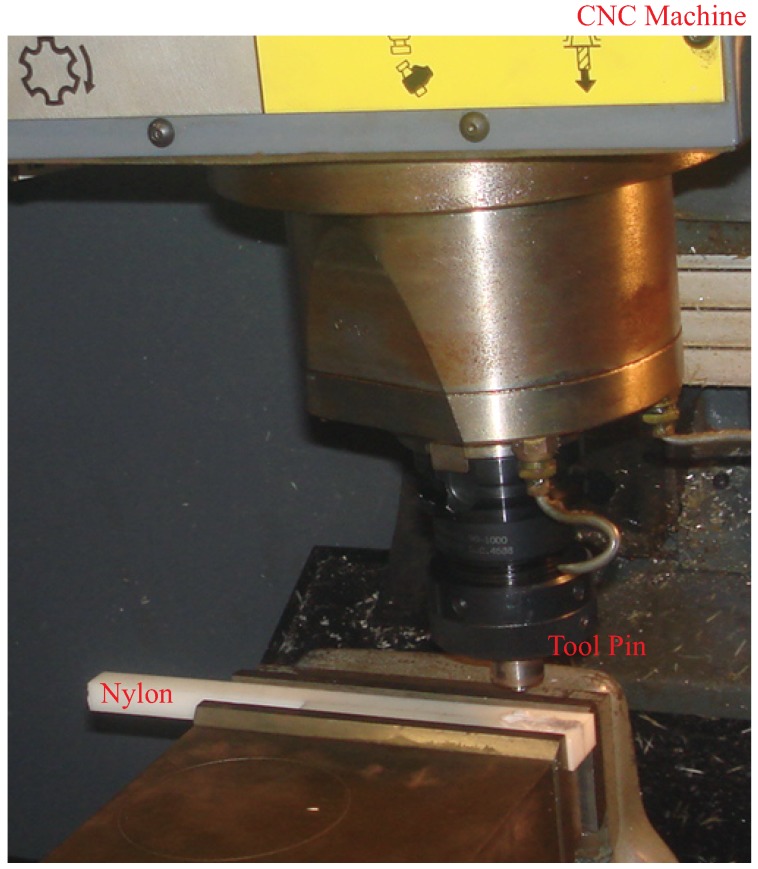
Equipment for friction stir processing polymer composites.

**Figure 13:**
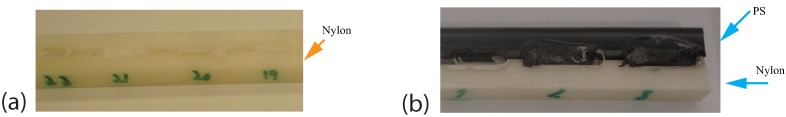
Friction stir processed composites; (a) nylon-nylon laminate, (b) nylon-polystyrene laminate.

Polymers have much lower processing temperatures than metals. The facilities for implementing FSP could be much simpler and less expensive. As shown in Figure 12, the experimental installation for the friction stir processing just includes the CNC machine, the tool pin connected to the spindle, and the specimen stage. Materials to be processed are fixed by the clamper. Friction stir processing of polymer composites was performed successfully. The polymeric materials used are particle filled plastics such as polystyrene (PS) and nylon. Figure 13(a) shows the nylon-nylon composite laminate, while nylon-polystyrene laminate is illustrated in Figure 13(b). Thermoplastics such as Polypropylene have been treated by friction stir processing [96,97]. It is noted that FSP polymers and polymeric composites are still much less studied than FSP metals and metallic composites [98]. There are some concerns related to friction stir processing polymers because the rotation of tool pin and the severe plastic flow in the nugget zone could break macromolecular chains and change the properties of the materials.

### 4.3. Issues on the Challenges and Improvements Needed

**Figure 14:**
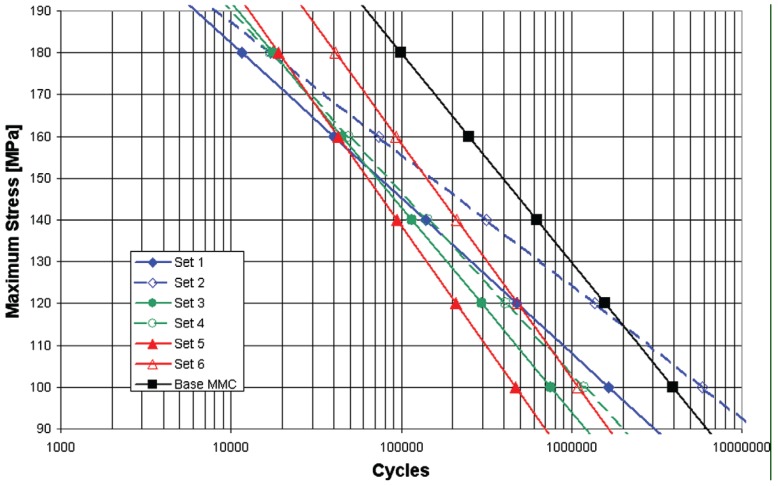
50% failure probability curves of friction stir processed 22 vol % Al_2_O_3_ particle reinforced AA6061 composites under different processing conditions (after Minak *et al.* [99]).

**Figure 15:**
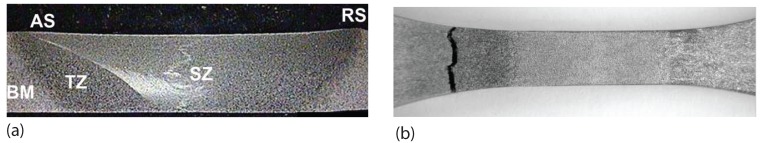
Macroscopic images of the friction stir processed 22 vol % Al_2_O_3_ particle reinforced AA6061 composite showing: (a) the base material (BM), the transition zone (TZ), the stir zone (SZ), the advancing side (AS), and the retreating side (RS); (b) typical location of fatigue fracture in the transition zone (TZ) (after Minak *et al.* [99]).

There are some challenges associated with friction stir processing approach. Although, friction stir processing has many advantages over various other materials processing technologies, the fact is that the joint efficiency defined as the ratio between the ultimate tensile strength of the welded joint and the one of the base material has hardly reached 80% [62]. This is an indication that the weld strength is still low. In addition to the static tensile strength, the fatigue property of FSP composites should also be improved. Minak *et al.* [99] have recently studied the fatigue properties of friction stir processed particulate reinforced aluminium matrix composites and made extensive statistical analysis. Failure probability curves were generated as shown in Figure 14. The composite material specimens with the set numbers from 1 to 6 have the same composition, but they were processed under different conditions. The processing parameters are given in Table 1. The statistical analysis results in Figure 14 showed that FSP specimens with different processing parameters had worse fatigue behavior than that of the base composite material. The different microstructural homogeneity in the transition from the base to the FSP zone is considered as the major reason for the degradation of fatigue property. As shown in Figure 15(a), the transition in microstructural homogeneity of the composite material generated by FSP can obviously be seen. The transition zone (TZ) seems the weakest part of the composite material because fatigue fracture always occurs in this zone for the specimens with the set numbers from 2 to 6. The macroscopic view of a typical fatigue-fractured specimen is shown in Figure 15(b).

Friction stir tool wear is another challenge problem remaining to be solved. This problem may not be so severe in FSP pure alloys without reinforcements. But it is serious during the processing of particle reinforced metal matrix composites (MMCs). In FSP soft metals without reinforcement particles such as aluminum alloys, though frictional heat is generated [100], no obvious tool consumption was created [101]. That is, no significant friction-induced wear occurs. The reason is that the stir zone consists of dynamically recrystallized (DRX) material which creates a solid-state flow regime where friction is minimal [102,103]. Therefore, the rotating pin tool is surrounded by a quasi-hydrodynamic lubricating layer composed of the DRX material [104,105,106].

**Table 1:** Parameters used for the FSP (data source: [99]).

Specimen set	1	2	3	4	5	6
Rotation rate (rpm)	630	630	630	880	880	880
Tool speed (mm/min)	115	170	260	115	170	260

**Figure 16:**
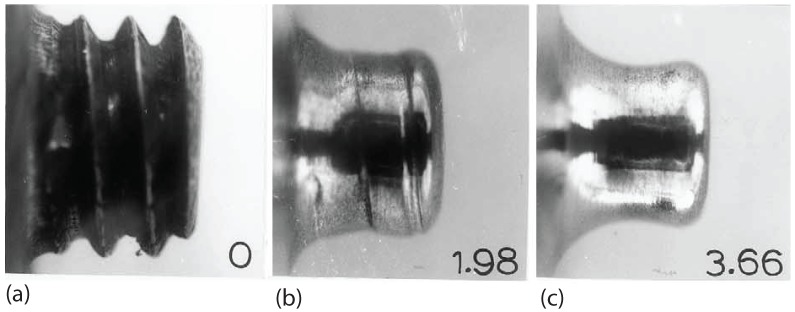
Macroscopic images showing the wear of friction stir processing tool (after Prado *et al.* [58]); (a) the screw tool pin before FSP, (b) after traveling 1.98 m, (c) after traveling 3.66 m.

Friction stir processing metal matrix composites (MMCs) can exhibit considerable tool consumption as shown in Figure 16 [58]. The tool wear was examined in friction stir processing 20 vol % Al_2_O_3_/AA6061 at the rotation rate of 1000 rpm and the traveling speed of 9 mm/s. Initially, the tool was in the screw shape as shown in Figure 16(a). After being used for FSP, the screw part disappeared, which means that the consumption of the tool occurred. When the tool was in service and traveled 1.96 m, the tool end was rounded up as shown in Figure 16(b). If the tool was further used, the surface wear continued. Figure 16(c) shows when the traveling distance reached 3.66 m, necking due to the increased wear was observed. In order to reduced the wear, cubic boron nitride and tungsten rhenium alloys may be used as tool materials, but they are extremely expensive and difficult to shape.

## 5. Summary and Conclusions

Friction stir processing (FSP) forces materials flow at the temperature lower than the melting temperature. Materials are extruded, forged, consolidated and cooled under hydrostatic pressure conditions. Thus some of the properties, for example, hardness is high for FSP materials. The primary research on friction stir processing focuses on aluminum alloys. Applying this technology for processing other alloys and materials including stainless steels, magnesium, titanium, and copper are also studied. This technology has found applications in modifying the microstructure of reinforced metal matrix composite materials. It is also used in processing polymeric composite materials. It is possible to transfer FSP technology into energy materials field for example, for fusion reactor materials including vanadium alloy joining and processing. There are some major challenges for improving the FSP technology. One is the tool wear in processing reinforced composite materials. The other challenge is how to increase the joining strength and improve the fatigue property of FSP composite materials.
